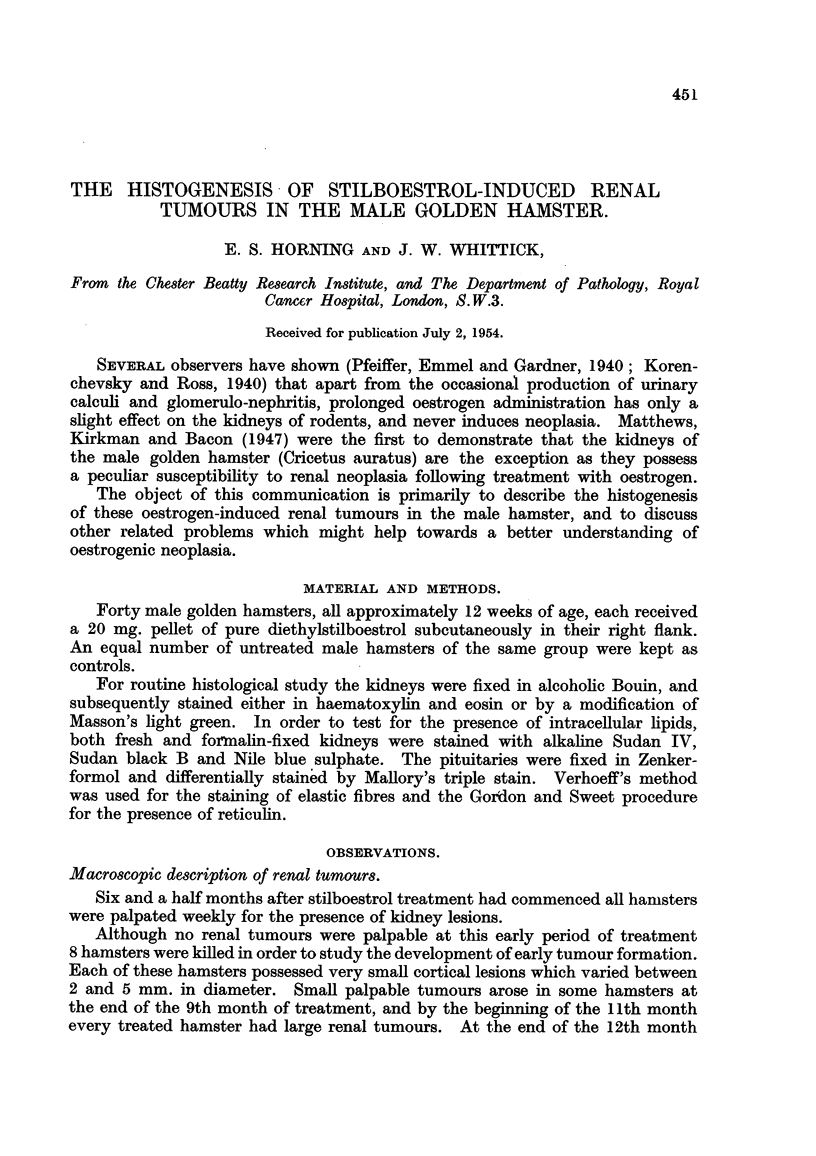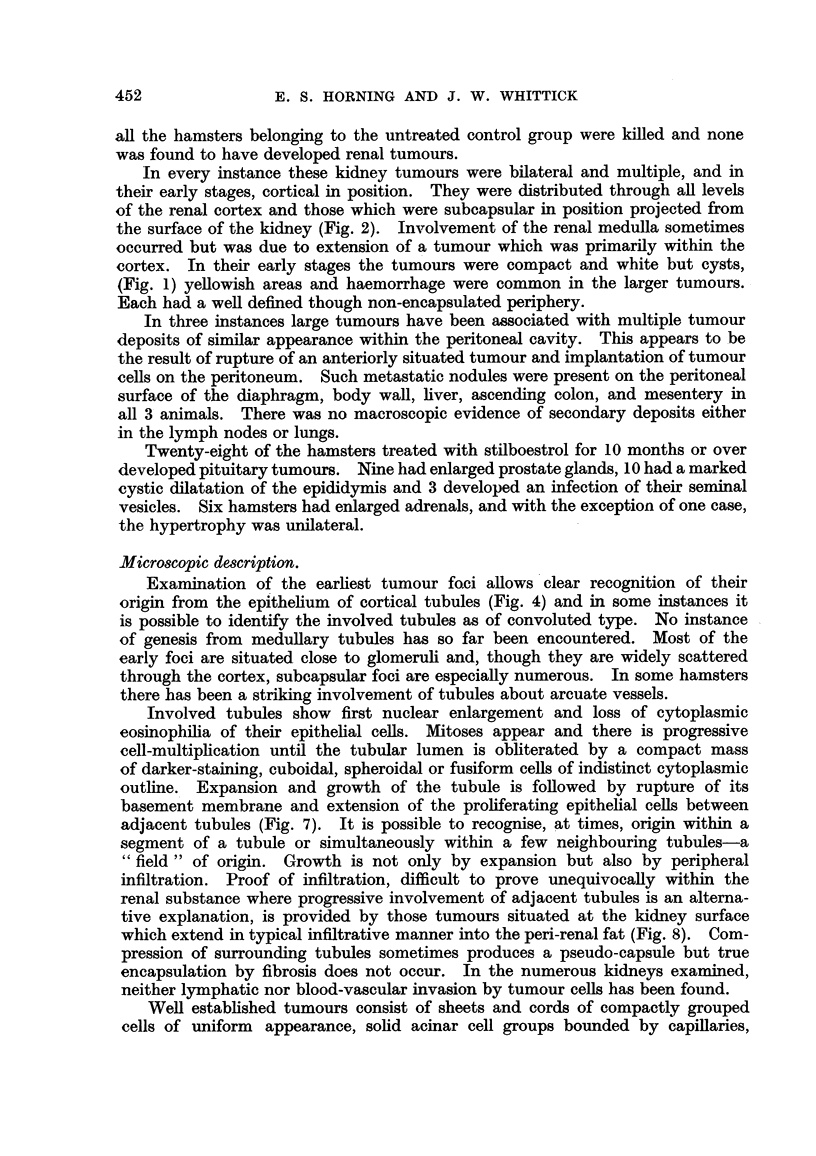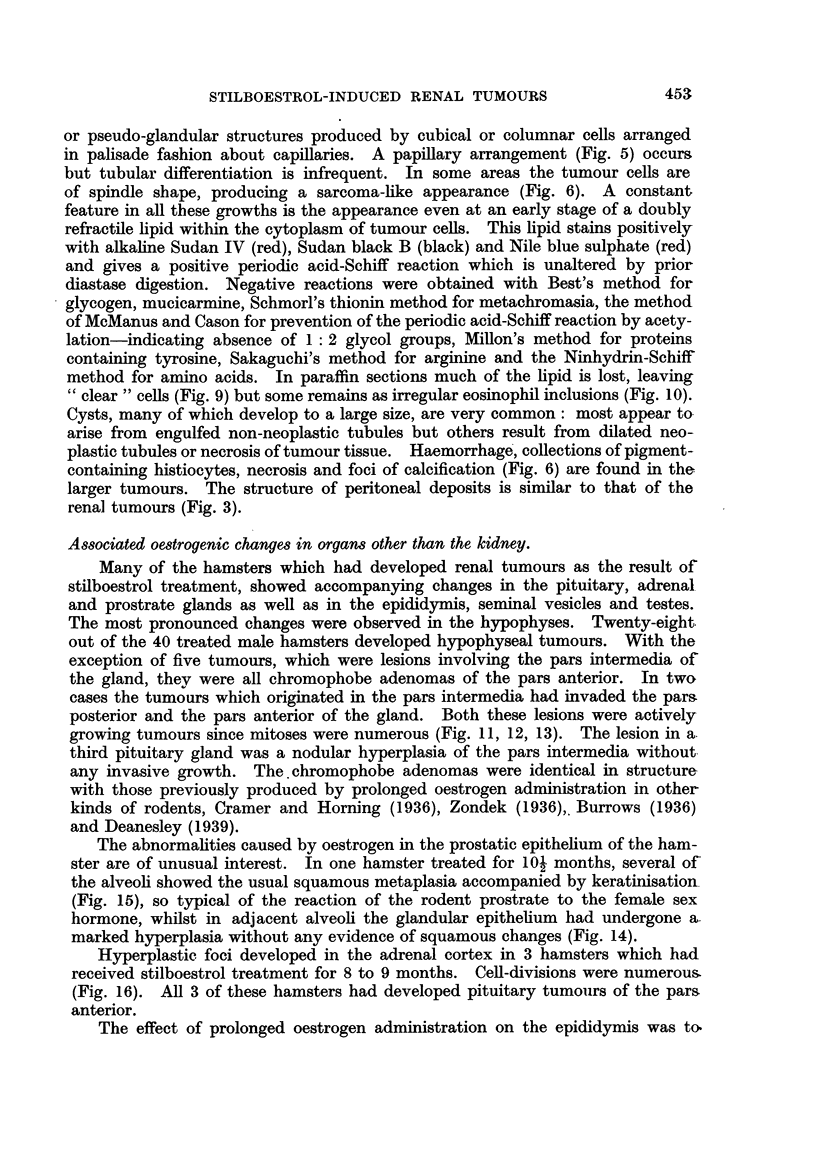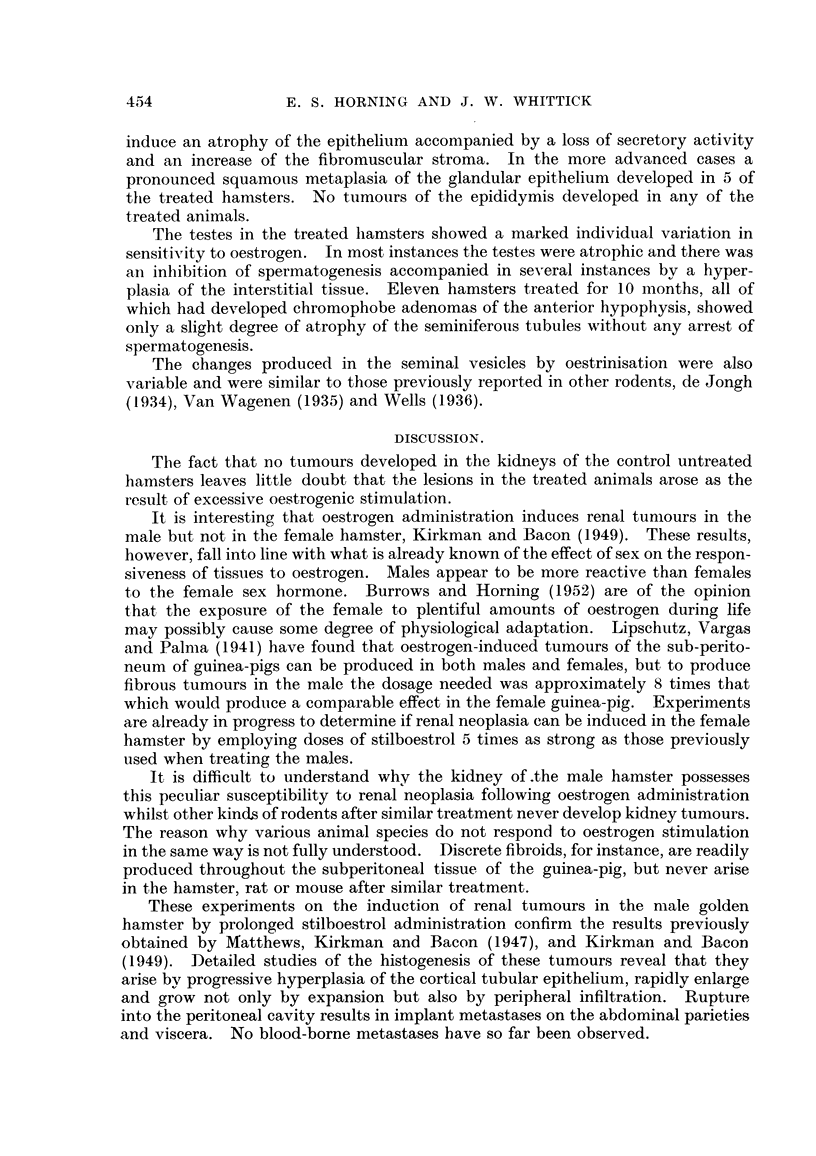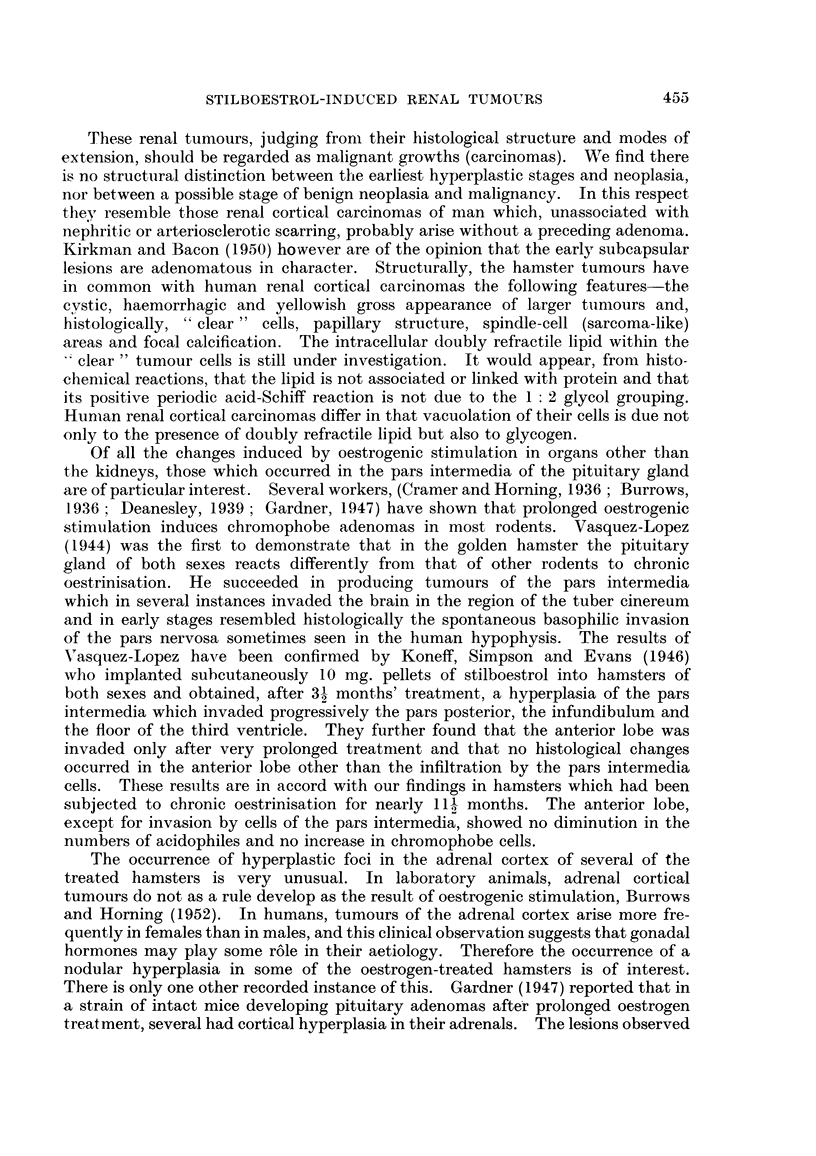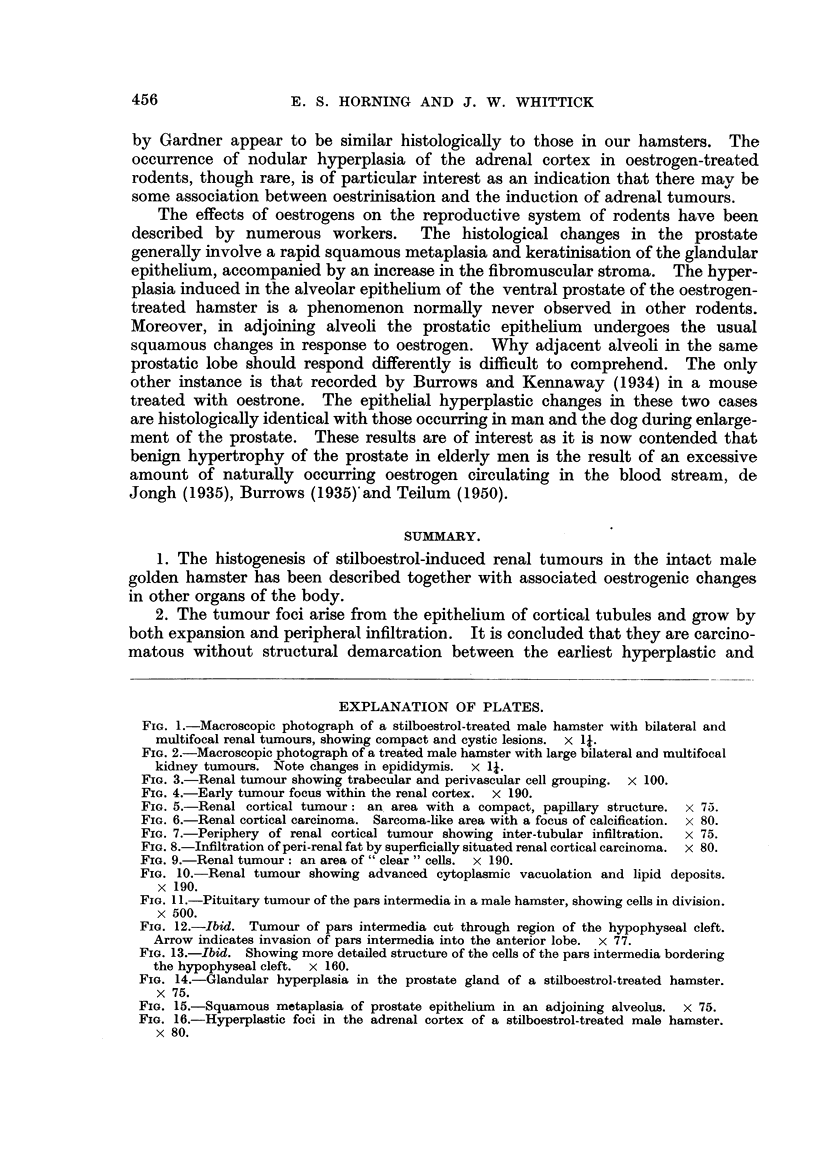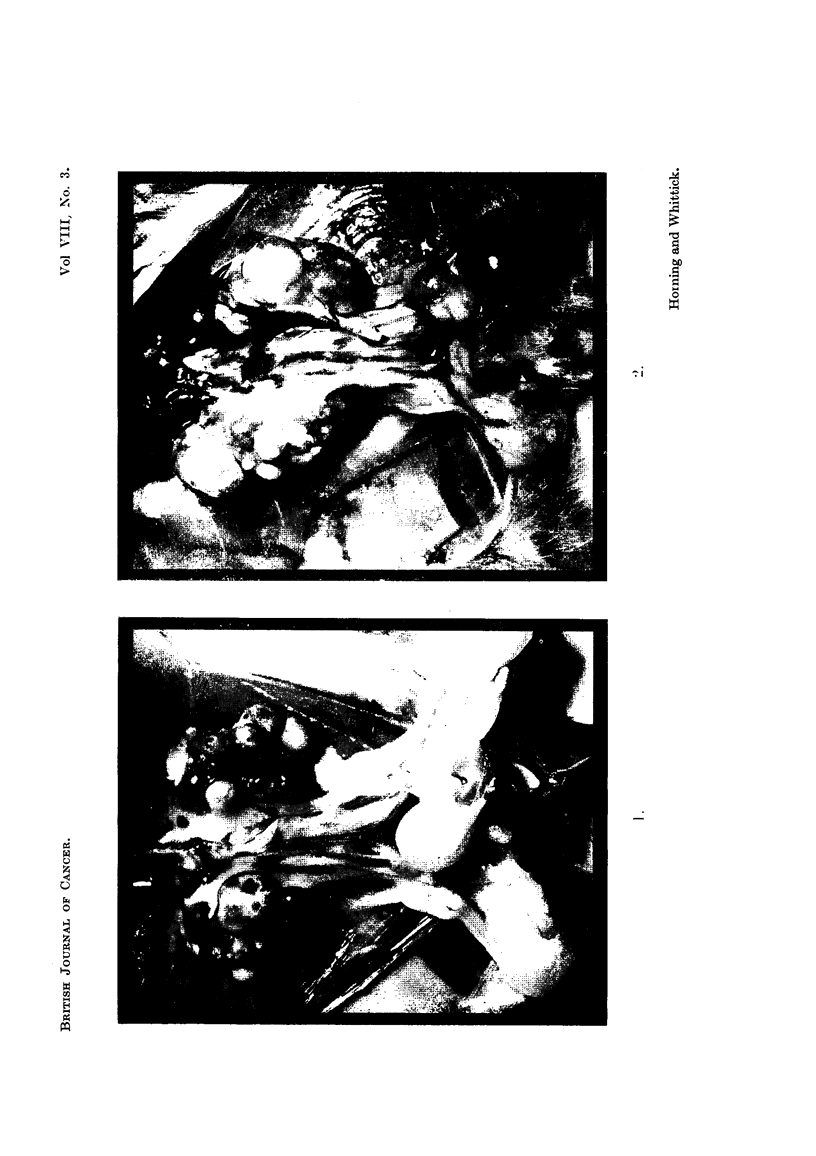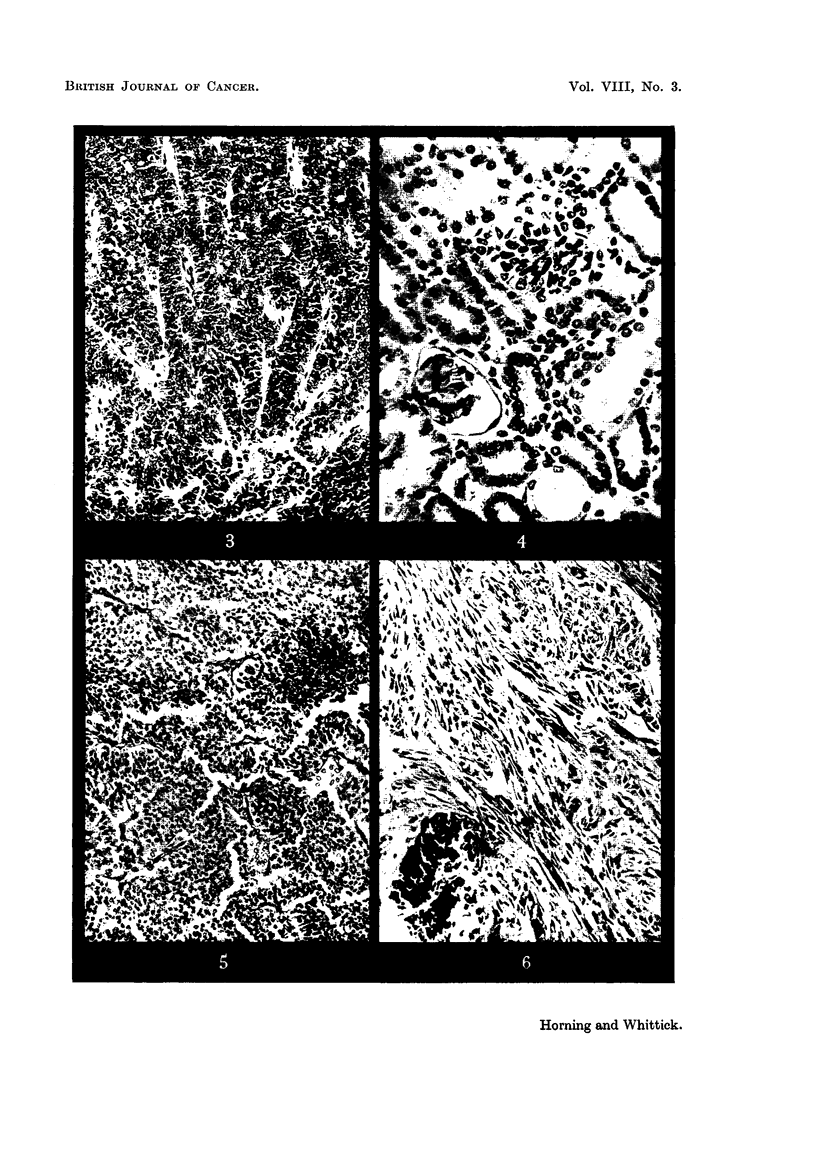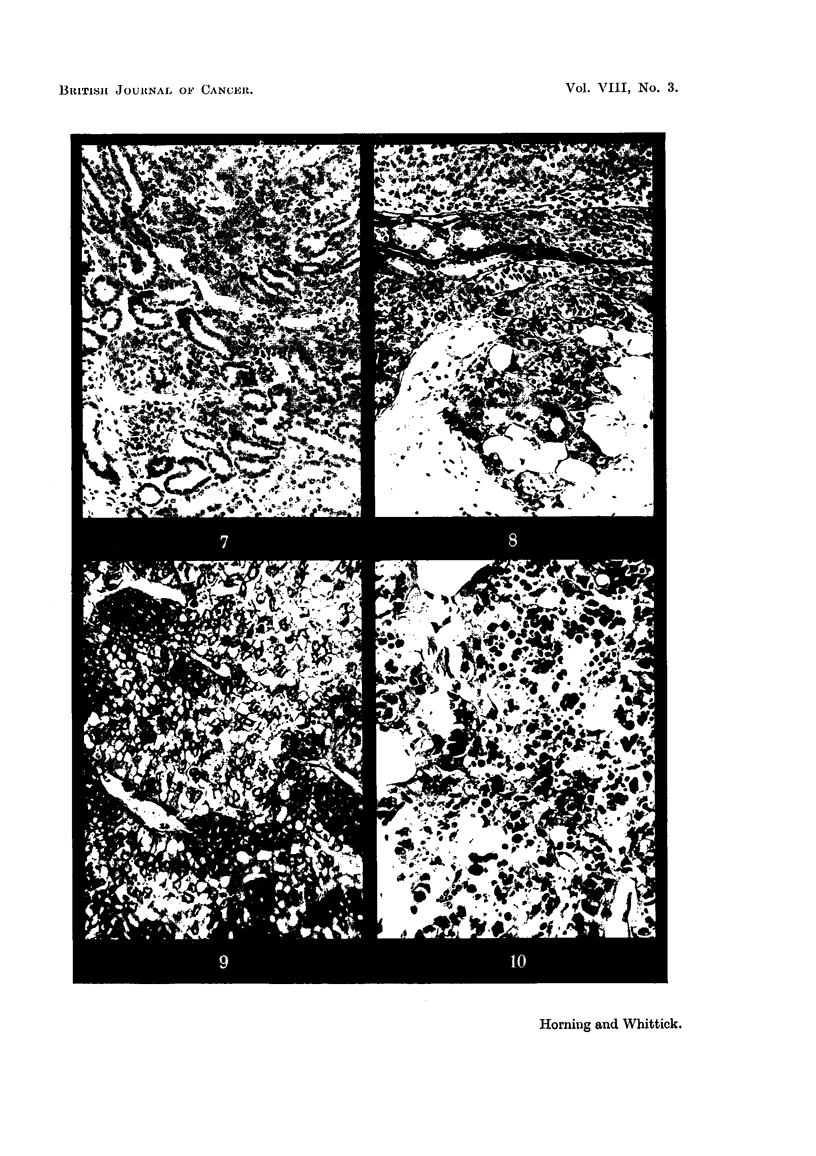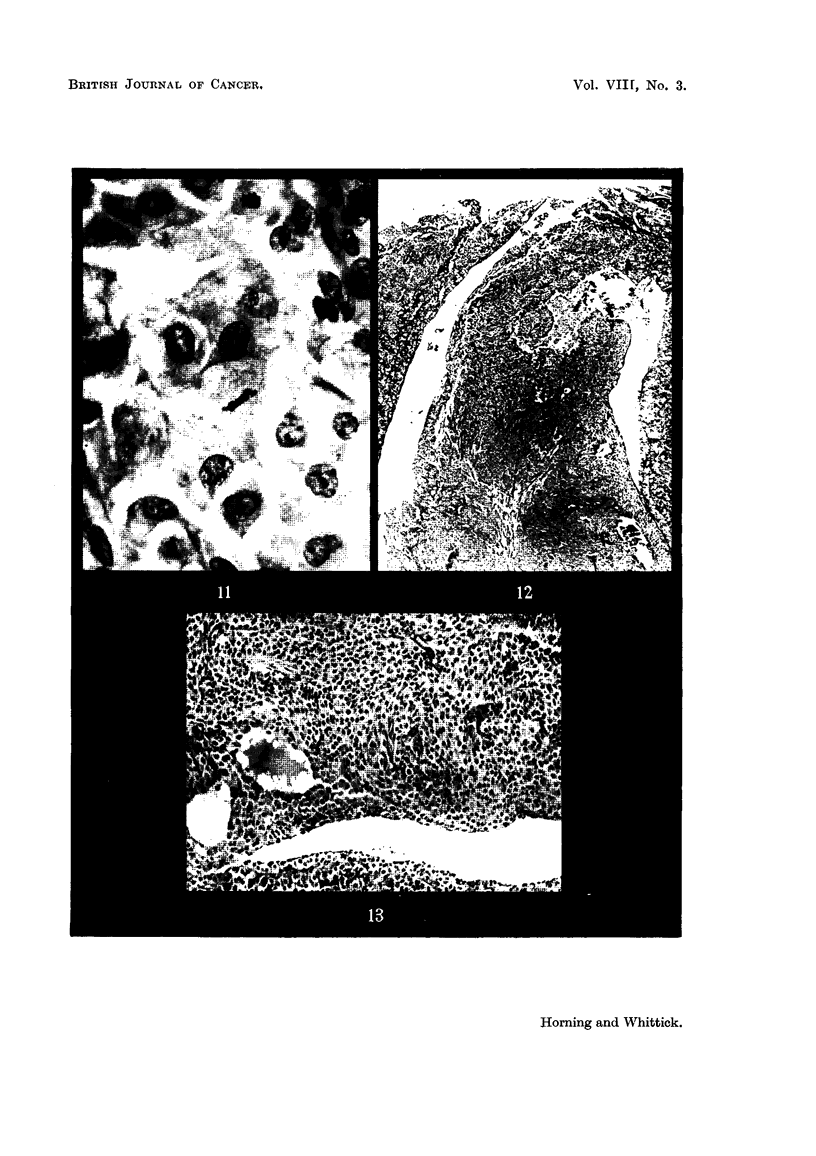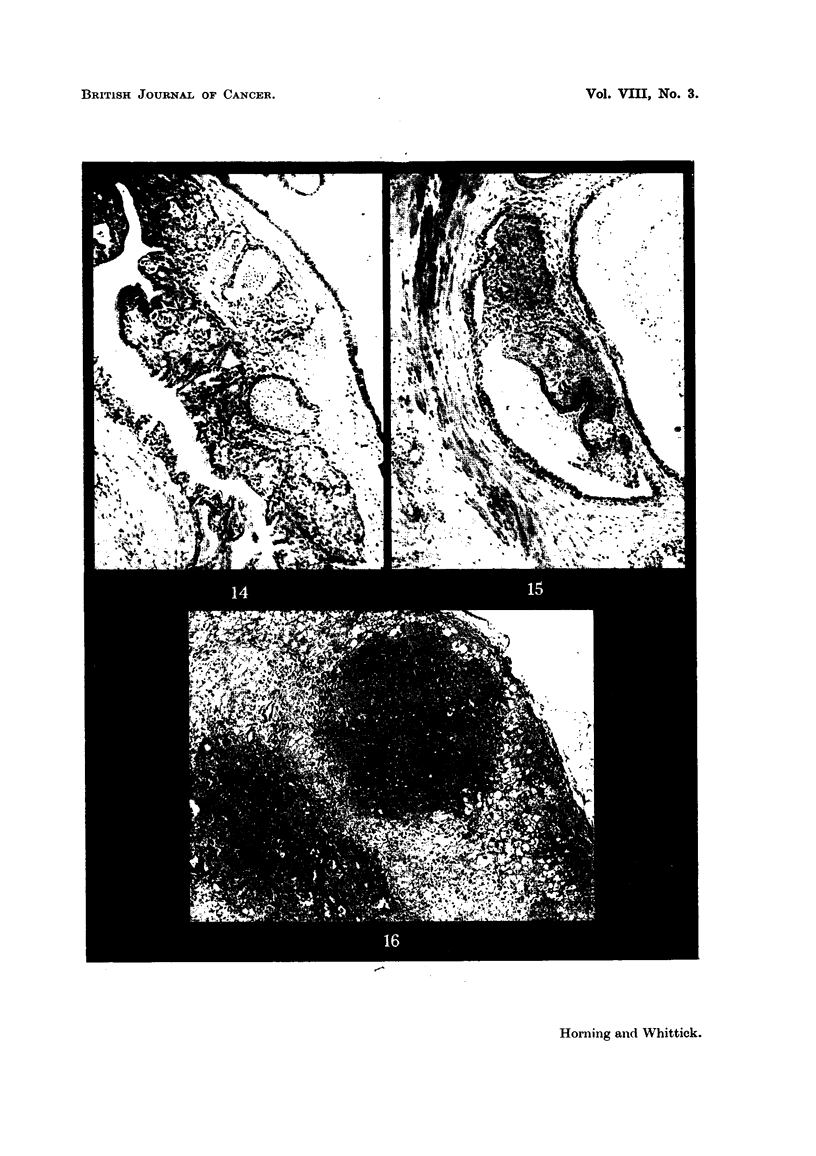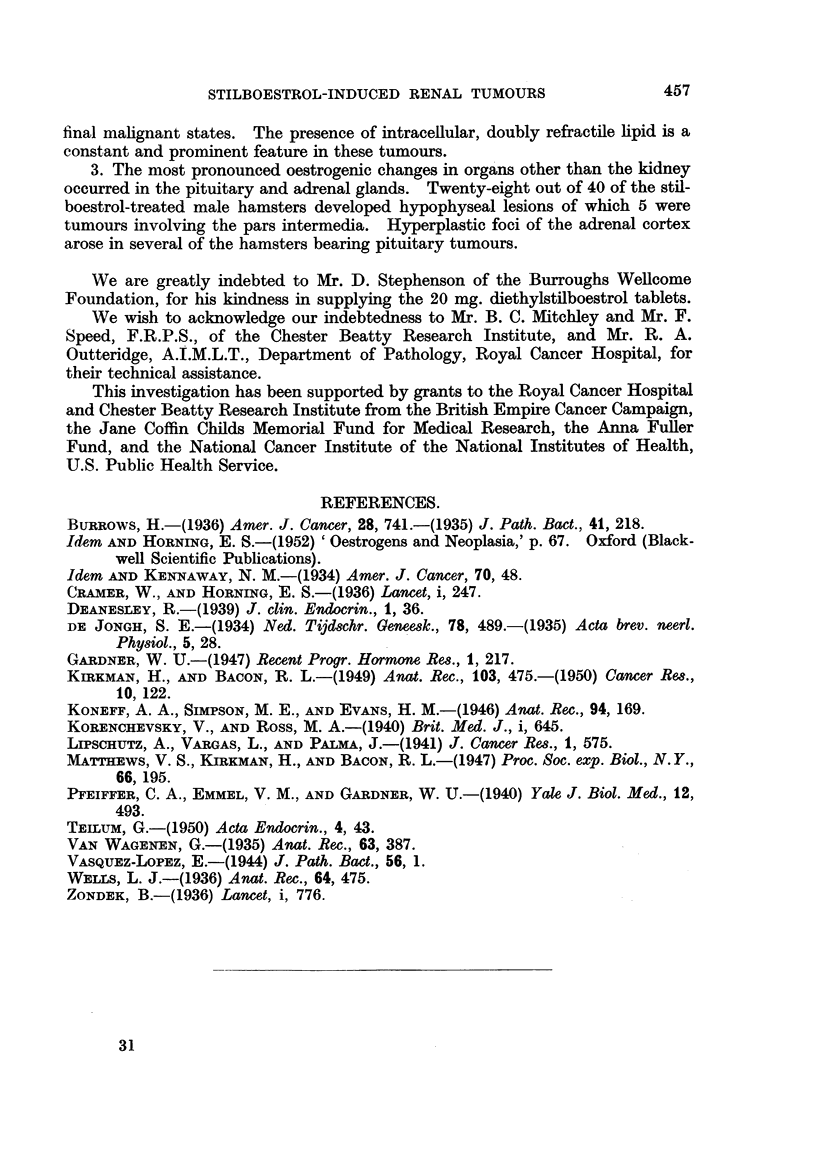# The Histogenesis of Stilboestrol-Induced Renal Tumours in the Male Golden Hamster

**DOI:** 10.1038/bjc.1954.48

**Published:** 1954-09

**Authors:** E. S. Horning, J. W. Whittick

## Abstract

**Images:**


### THE HISTOGENESIS OF STILBOESTROL-INDUCED RENAL TUMOURS IN THE MALE GOLDEN HAMSTER.

E. S. HORNING AND J. W. WHITTICK,

*From the Chester Beatty Research Institute, and The Department of Pathology, Royal Cancer Hospital, London, S.W.3.*

Received for publication July 2, 1954.

SEVERAL observers have shown (Pfeiffer, Emmel and Gardner, 1940; Korenchevsky and Ross, 1940) that apart from the occasional production of urinary calculi and glomerulo-nephritis, prolonged oestrogen administration has only a slight effect on the kidneys of rodents, and never induces neoplasia. Matthews, Kirkman and Bacon (1947) were the first to demonstrate that the kidneys of the male golden hamster (Cricetus auratus) are the exception as they possess a peculiar susceptibility to renal neoplasia following treatment with oestrogen.

The object of this communication is primarily to describe the histogenesis of these oestrogen-induced renal tumours in the male hamster, and to discuss other related problems which might help towards a better understanding of oestrogenic neoplasia.

### MATERIAL AND METHODS.

Forty male golden hamsters, all approximately 12 weeks of age, each received a 20 mg. pellet of pure diethylstilboestrol subcutaneously in their right flank. An equal number of untreated male hamsters of the same group were kept as controls.

For routine histological study the kidneys were fixed in alcoholic Bouin, and subsequently stained either in haematoxylin and eosin or by a modification of Masson's light green. In order to test for the presence of intracellular lipids, both fresh and formalin-fixed kidneys were stained with alkaline Sudan IV, Sudan black B and Nile blue sulphate. The pituitaries were fixed in Zenker-formol and differentially stained by Mallory's triple stain. Verhoeff's method was used for the staining of elastic fibres and the Gordon and Sweet procedure for the presence of reticulin.

### OBSERVATIONS.

### *Macroscopic description of renal tumours.*

Six and a half months after stilboestrol treatment had commenced all hamsters were palpated weekly for the presence of kidney lesions.

Although no renal tumours were palpable at this early period of treatment 8 hamsters were killed in order to study the development of early tumour formation. Each of these hamsters possessed very small cortical lesions which varied between 2 and 5 mm. in diameter. Small palpable tumours arose in some hamsters at the end of the 9th month of treatment, and by the beginning of the 11th month every treated hamster had large renal tumours. At the end of the 12th month all the hamsters belonging to the untreated control group were killed and none was found to have developed renal tumours.

In every instance these kidney tumours were bilateral and multiple, and in their early stages, cortical in position. They were distributed through all levels of the renal cortex and those which were subcapsular in position projected from the surface of the kidney (Fig. 2). Involvement of the renal medulla sometimes occurred but was due to extension of a tumour which was primarily within the cortex. In their early stages the tumours were compact and white but cysts, (Fig. 1) yellowish areas and haemorrhage were common in the larger tumours. Each had a well defined though non-encapsulated periphery.

In three instances large tumours have been associated with multiple tumour deposits of similar appearance within the peritoneal cavity. This appears to be the result of rupture of an anteriorly situated tumour and implantation of tumour cells on the peritoneum. Such metastatic nodules were present on the peritoneal surface of the diaphragm, body wall, liver, ascending colon, and mesentery in all 3 animals. There was no macroscopic evidence of secondary deposits either in the lymph nodes or lungs.

Twenty-eight of the hamsters treated with stilboestrol for 10 months or over developed pituitary tumours. Nine had enlarged prostate glands, 10 had a marked cystic dilatation of the epididymis and 3 developed an infection of their seminal vesicles. Six hamsters had enlarged adrenals, and with the exception of one case, the hypertrophy was unilateral.

*Microscopic description.*

Examination of the earliest tumour foci allows clear recognition of their origin from the epithelium of cortical tubules (Fig. 4) and in some instances it is possible to identify the involved tubules as of convoluted type. No instance of genesis from medullary tubules has so far been encountered. Most of the early foci are situated close to glomeruli and, though they are widely scattered through the cortex, subcapsular foci are especially numerous. In some hamsters there has been a striking involvement of tubules about arcuate vessels.

Involved tubules show first nuclear enlargement and loss of cytoplasmic eosinophilia of their epithelial cells. Mitoses appear and there is progressive cell-multiplication until the tubular lumen is obliterated by a compact mass of darker-staining, cuboidal, spheroidal or fusiform cells of indistinct cytoplasmic outline. Expansion and growth of the tubule is followed by rupture of its basement membrane and extension of the proliferating epithelial cells between adjacent tubules (Fig. 7). It is possible to recognise, at times, origin within a segment of a tubule or simultaneously within a few neighbouring tubules—a " field " of origin. Growth is not only by expansion but also by peripheral infiltration. Proof of infiltration, difficult to prove unequivocally within the renal substance where progressive involvement of adjacent tubules is an alternative explanation, is provided by those tumours situated at the kidney surface which extend in typical infiltrative manner into the peri-renal fat (Fig. 8). Compression of surrounding tubules sometimes produces a pseudo-capsule but true encapsulation by fibrosis does not occur. In the numerous kidneys examined, neither lymphatic nor blood-vascular invasion by tumour cells has been found.

Well established tumours consist of sheets and cords of compactly grouped cells of uniform appearance, solid acinar cell groups bounded by capillaries, or pseudo-glandular structures produced by cubical or columnar cells arranged in palisade fashion about capillaries. A papillary arrangement (Fig. 5) occurs but tubular differentiation is infrequent. In some areas the tumour cells are of spindle shape, producing a sarcoma-like appearance (Fig. 6). A constant feature in all these growths is the appearance even at an early stage of a doubly refractile lipid within the cytoplasm of tumour cells. This lipid stains positively with alkaline Sudan IV (red), Sudan black B (black) and Nile blue sulphate (red) and gives a positive periodic acid-Schiff reaction which is unaltered by prior diastase digestion. Negative reactions were obtained with Best's method for glycogen, mucicarmine, Schmorl's thionin method for metachromasia, the method of McManus and Cason for prevention of the periodic acid-Schiff reaction by acetylation—indicating absence of 1 : 2 glycol groups, Millon's method for proteins containing tyrosine, Sakaguchi's method for arginine and the Ninhydrin-Schiff method for amino acids. In paraffin sections much of the lipid is lost, leaving " clear " cells (Fig. 9) but some remains as irregular eosinophil inclusions (Fig. 10). Cysts, many of which develop to a large size, are very common : most appear to arise from engulfed non-neoplastic tubules but others result from dilated neoplastic tubules or necrosis of tumour tissue. Haemorrhage, collections of pigment-containing histiocytes, necrosis and foci of calcification (Fig. 6) are found in the larger tumours. The structure of peritoneal deposits is similar to that of the renal tumours (Fig. 3).

*Associated oestrogenic changes in organs other than the kidney.*

Many of the hamsters which had developed renal tumours as the result of stilboestrol treatment, showed accompanying changes in the pituitary, adrenal and prostrate glands as well as in the epididymis, seminal vesicles and testes. The most pronounced changes were observed in the hypophyses. Twenty-eight out of the 40 treated male hamsters developed hypophyseal tumours. With the exception of five tumours, which were lesions involving the pars intermedia of the gland, they were all chromophobe adenomas of the pars anterior. In two cases the tumours which originated in the pars intermedia had invaded the pars posterior and the pars anterior of the gland. Both these lesions were actively growing tumours since mitoses were numerous (Fig. 11, 12, 13). The lesion in a third pituitary gland was a nodular hyperplasia of the pars intermedia without any invasive growth. The chromophobe adenomas were identical in structure with those previously produced by prolonged oestrogen administration in other kinds of rodents, Cramer and Horning (1936), Zondek (1936), Burrows (1936) and Deanesley (1939).

The abnormalities caused by oestrogen in the prostatic epithelium of the hamster are of unusual interest. In one hamster treated for 10½ months, several of the alveoli showed the usual squamous metaplasia accompanied by keratinisation (Fig. 15), so typical of the reaction of the rodent prostrate to the female sex hormone, whilst in adjacent alveoli the glandular epithelium had undergone a marked hyperplasia without any evidence of squamous changes (Fig. 14).

Hyperplastic foci developed in the adrenal cortex in 3 hamsters which had received stilboestrol treatment for 8 to 9 months. Cell-divisions were numerous (Fig. 16). All 3 of these hamsters had developed pituitary tumours of the pars anterior.

The effect of prolonged oestrogen administration on the epididymis was to induce an atrophy of the epithelium accompanied by a loss of secretory activity and an increase of the fibromuscular stroma. In the more advanced cases a pronounced squamous metaplasia of the glandular epithelium developed in 5 of the treated hamsters. No tumours of the epididymis developed in any of the treated animals.

The testes in the treated hamsters showed a marked individual variation in sensitivity to oestrogen. In most instances the testes were atrophic and there was an inhibition of spermatogenesis accompanied in several instances by a hyperplasia of the interstitial tissue. Eleven hamsters treated for 10 months, all of which had developed chromophobe adenomas of the anterior hypophysis, showed only a slight degree of atrophy of the seminiferous tubules without any arrest of spermatogenesis.

The changes produced in the seminal vesicles by oestrinisation were also variable and were similar to those previously reported in other rodents, de Jongh (1934), Van Wagenen (1935) and Wells (1936).

### DISCUSSION.

The fact that no tumours developed in the kidneys of the control untreated hamsters leaves little doubt that the lesions in the treated animals arose as the result of excessive oestrogenic stimulation.

It is interesting that oestrogen administration induces renal tumours in the male but not in the female hamster, Kirkman and Bacon (1949). These results, however, fall into line with what is already known of the effect of sex on the responsiveness of tissues to oestrogen. Males appear to be more reactive than females to the female sex hormone. Burrows and Horning (1952) are of the opinion that the exposure of the female to plentiful amounts of oestrogen during life may possibly cause some degree of physiological adaptation. Lipschutz, Vargas and Palma (1941) have found that oestrogen-induced tumours of the sub-peritoneum of guinea-pigs can be produced in both males and females, but to produce fibrous tumours in the male the dosage needed was approximately 8 times that which would produce a comparable effect in the female guinea-pig. Experiments are already in progress to determine if renal neoplasia can be induced in the female hamster by employing doses of stilboestrol 5 times as strong as those previously used when treating the males.

It is difficult to understand why the kidney of .the male hamster possesses this peculiar susceptibility to renal neoplasia following oestrogen administration whilst other kinds of rodents after similar treatment never develop kidney tumours. The reason why various animal species do not respond to oestrogen stimulation in the same way is not fully understood. Discrete fibroids, for instance, are readily produced throughout the subperitoneal tissue of the guinea-pig, but never arise in the hamster, rat or mouse after similar treatment.

These experiments on the induction of renal tumours in the male golden hamster by prolonged stilboestrol administration confirm the results previously obtained by Matthews, Kirkman and Bacon (1947), and Kirkman and Bacon (1949). Detailed studies of the histogenesis of these tumours reveal that they arise by progressive hyperplasia of the cortical tubular epithelium, rapidly enlarge and grow not only by expansion but also by peripheral infiltration. Rupture into the peritoneal cavity results in implant metastases on the abdominal parieties and viscera. No blood-borne metastases have so far been observed.

These renal tumours, judging from their histological structure and modes of extension, should be regarded as malignant growths (carcinomas). We find there is no structural distinction between the earliest hyperplastic stages and neoplasia, nor between a possible stage of benign neoplasia and malignancy. In this respect they resemble those renal cortical carcinomas of man which, unassociated with nephritic or arteriosclerotic scarring, probably arise without a preceding adenoma. Kirkman and Bacon (1950) however are of the opinion that the early subcapsular lesions are adenomatous in character. Structurally, the hamster tumours have in common with human renal cortical carcinomas the following features—the cystic, haemorrhagic and yellowish gross appearance of larger tumours and, histologically, " clear " cells, papillary structure, spindle-cell (sarcoma-like) areas and focal calcification. The intracellular doubly refractile lipid within the " clear " tumour cells is still under investigation. It would appear, from histochemical reactions, that the lipid is not associated or linked with protein and that its positive periodic acid-Schiff reaction is not due to the 1 : 2 glycol grouping. Human renal cortical carcinomas differ in that vacuolation of their cells is due not only to the presence of doubly refractile lipid but also to glycogen.

Of all the changes induced by oestrogenic stimulation in organs other than the kidneys, those which occurred in the pars intermedia of the pituitary gland are of particular interest. Several workers, (Cramer and Horning, 1936 ; Burrows, 1936 ; Deanesley, 1939 ; Gardner, 1947) have shown that prolonged oestrogenic stimulation induces chromophobe adenomas in most rodents. Vasquez-Lopez (1944) was the first to demonstrate that in the golden hamster the pituitary gland of both sexes reacts differently from that of other rodents to chronic oestrinisation. He succeeded in producing tumours of the pars intermedia which in several instances invaded the brain in the region of the tuber cinereum and in early stages resembled histologically the spontaneous basophilic invasion of the pars nervosa sometimes seen in the human hypophysis. The results of Vasquez-Lopez have been confirmed by Koneff, Simpson and Evans (1946) who implanted subcutaneously 10 mg. pellets of stilboestrol into hamsters of both sexes and obtained, after $3\frac{1}{2}$ months' treatment, a hyperplasia of the pars intermedia which invaded progressively the pars posterior, the infundibulum and the floor of the third ventricle. They further found that the anterior lobe was invaded only after very prolonged treatment and that no histological changes occurred in the anterior lobe other than the infiltration by the pars intermedia cells. These results are in accord with our findings in hamsters which had been subjected to chronic oestrinisation for nearly $11\frac{1}{2}$ months. The anterior lobe, except for invasion by cells of the pars intermedia, showed no diminution in the numbers of acidophiles and no increase in chromophobe cells.

The occurrence of hyperplastic foci in the adrenal cortex of several of the treated hamsters is very unusual. In laboratory animals, adrenal cortical tumours do not as a rule develop as the result of oestrogenic stimulation, Burrows and Horning (1952). In humans, tumours of the adrenal cortex arise more frequently in females than in males, and this clinical observation suggests that gonadal hormones may play some rôle in their aetiology. Therefore the occurrence of a nodular hyperplasia in some of the oestrogen-treated hamsters is of interest. There is only one other recorded instance of this. Gardner (1947) reported that in a strain of intact mice developing pituitary adenomas after prolonged oestrogen treatment, several had cortical hyperplasia in their adrenals. The lesions observed by Gardner appear to be similar histologically to those in our hamsters. The occurrence of nodular hyperplasia of the adrenal cortex in oestrogen-treated rodents, though rare, is of particular interest as an indication that there may be some association between oestrinisation and the induction of adrenal tumours.

The effects of oestrogens on the reproductive system of rodents have been described by numerous workers. The histological changes in the prostate generally involve a rapid squamous metaplasia and keratinisation of the glandular epithelium, accompanied by an increase in the fibromuscular stroma. The hyperplasia induced in the alveolar epithelium of the ventral prostate of the oestrogen-treated hamster is a phenomenon normally never observed in other rodents. Moreover, in adjoining alveoli the prostatic epithelium undergoes the usual squamous changes in response to oestrogen. Why adjacent alveoli in the same prostatic lobe should respond differently is difficult to comprehend. The only other instance is that recorded by Burrows and Kennaway (1934) in a mouse treated with oestrone. The epithelial hyperplastic changes in these two cases are histologically identical with those occurring in man and the dog during enlargement of the prostate. These results are of interest as it is now contended that benign hypertrophy of the prostate in elderly men is the result of an excessive amount of naturally occurring oestrogen circulating in the blood stream, de Jongh (1935), Burrows (1935) and Teilum (1950).

### SUMMARY.

1. The histogenesis of stilboestrol-induced renal tumours in the intact male golden hamster has been described together with associated oestrogenic changes in other organs of the body.

2. The tumour foci arise from the epithelium of cortical tubules and grow by both expansion and peripheral infiltration. It is concluded that they are carcinomatous without structural demarcation between the earliest hyperplastic and

### EXPLANATION OF PLATES.

FIG. 1.—Macroscopic photograph of a stilboestrol-treated male hamster with bilateral and multifocal renal tumours, showing compact and cystic lesions. × 1¼.
FIG. 2.—Macroscopic photograph of a treated male hamster with large bilateral and multifocal kidney tumours. Note changes in epididymis. × 1¼.
FIG. 3.—Renal tumour showing trabecular and perivascular cell grouping. × 100.
FIG. 4.—Early tumour focus within the renal cortex. × 190.
FIG. 5.—Renal cortical tumour: an area with a compact, papillary structure. × 75.
FIG. 6.—Renal cortical carcinoma. Sarcoma-like area with a focus of calcification. × 80.
FIG. 7.—Periphery of renal cortical tumour showing inter-tubular infiltration. × 75.
FIG. 8.—Infiltration of peri-renal fat by superficially situated renal cortical carcinoma. × 80.
FIG. 9.—Renal tumour: an area of " clear " cells. × 190.
FIG. 10.—Renal tumour showing advanced cytoplasmic vacuolation and lipid deposits. × 190.
FIG. 11.—Pituitary tumour of the pars intermedia in a male hamster, showing cells in division. × 500.
FIG. 12.—*Ibid.* Tumour of pars intermedia cut through region of the hypophyseal cleft. Arrow indicates invasion of pars intermedia into the anterior lobe. × 77.
FIG. 13.—*Ibid.* Showing more detailed structure of the cells of the pars intermedia bordering the hypophyseal cleft. × 160.
FIG. 14.—Glandular hyperplasia in the prostate gland of a stilboestrol-treated hamster. × 75.
FIG. 15.—Squamous metaplasia of prostate epithelium in an adjoining alveolus. × 75.
FIG. 16.—Hyperplastic foci in the adrenal cortex of a stilboestrol-treated male hamster. × 80.

1.

2.

Horning and Whittick.

Horning and Whittick.

7 8 9 10

Horning and Whittick.

11

12

13

Horning and Whittick.

14

15

16

final malignant states. The presence of intracellular, doubly refractile lipid is a constant and prominent feature in these tumours.

3. The most pronounced oestrogenic changes in organs other than the kidney occurred in the pituitary and adrenal glands. Twenty-eight out of 40 of the stilboestrol-treated male hamsters developed hypophyseal lesions of which 5 were tumours involving the pars intermedia. Hyperplastic foci of the adrenal cortex arose in several of the hamsters bearing pituitary tumours.

We are greatly indebted to Mr. D. Stephenson of the Burroughs Wellcome Foundation, for his kindness in supplying the 20 mg. diethylstilboestrol tablets.

We wish to acknowledge our indebtedness to Mr. B. C. Mitchley and Mr. F. Speed, F.R.P.S., of the Chester Beatty Research Institute, and Mr. R. A. Outteridge, A.I.M.L.T., Department of Pathology, Royal Cancer Hospital, for their technical assistance.

This investigation has been supported by grants to the Royal Cancer Hospital and Chester Beatty Research Institute from the British Empire Cancer Campaign, the Jane Coffin Childs Memorial Fund for Medical Research, the Anna Fuller Fund, and the National Cancer Institute of the National Institutes of Health, U.S. Public Health Service.

### REFERENCES.

BURROWS, H.—(1936) *Amer. J. Cancer*, **28**, 741.—(1935) *J. Path. Bact.*, **41**, 218.

*Idem* AND HORNING, E. S.—(1952) ' Oestrogens and Neoplasia,' p. 67. Oxford (Blackwell Scientific Publications).

*Idem* AND KENNAWAY, N. M.—(1934) *Amer. J. Cancer*, **70**, 48.

CRAMER, W., AND HORNING, E. S.—(1936) *Lancet*, i, 247.

DEANESLEY, R.—(1939) *J. clin. Endocrin.*, **1**, 36.

DE JONGH, S. E.—(1934) *Ned. Tijdschr. Geneesk.*, **78**, 489.—(1935) *Acta brev. neerl. Physiol.*, **5**, 28.

GARDNER, W. U.—(1947) *Recent Progr. Hormone Res.*, **1**, 217.

KIRKMAN, H., AND BACON, R. L.—(1949) *Anat. Rec.*, **103**, 475.—(1950) *Cancer Res.*, **10**, 122.

KONEFF, A. A., SIMPSON, M. E., AND EVANS, H. M.—(1946) *Anat. Rec.*, **94**, 169.

KORENCHEVSKY, V., AND ROSS, M. A.—(1940) *Brit. Med. J.*, i, 645.

LIPSCHUTZ, A., VARGAS, L., AND PALMA, J.—(1941) *J. Cancer Res.*, **1**, 575.

MATTHEWS, V. S., KIRKMAN, H., AND BACON, R. L.—(1947) *Proc. Soc. exp. Biol., N.Y.*, **66**, 195.

PFEIFFER, C. A., EMMEL, V. M., AND GARDNER, W. U.—(1940) *Yale J. Biol. Med.*, **12**, 493.

TEILUM, G.—(1950) *Acta Endocrin.*, **4**, 43.

VAN WAGENEN, G.—(1935) *Anat. Rec.*, **63**, 387.

VASQUEZ-LOPEZ, E.—(1944) *J. Path. Bact.*, **56**, 1.

WELLS, L. J.—(1936) *Anat. Rec.*, **64**, 475.

ZONDEK, B.—(1936) *Lancet*, i, 776.